# Viruses in colorectal cancer

**DOI:** 10.1002/1878-0261.13100

**Published:** 2021-09-30

**Authors:** Luigi Marongiu, Heike Allgayer

**Affiliations:** ^1^ Department of Experimental Surgery – Cancer Metastasis Medical Faculty Mannheim Ruprecht‐Karls‐University of Heidelberg Mannheim Germany

**Keywords:** colorectal cancer, metastasis, phages, viruses

## Abstract

Increasing evidence suggests that microorganisms might represent at least highly interesting cofactors in colorectal cancer (CRC) oncogenesis and progression. Still, associated mechanisms, specifically in colonocytes and their microenvironmental interactions, are still poorly understood. Although, currently, at least seven viruses are being recognized as human carcinogens, only three of these – Epstein–Barr virus (EBV), human papillomavirus (HPV) and John Cunningham virus (JCV) – have been described, with varying levels of evidence, in CRC. In addition, cytomegalovirus (CMV) has been associated with CRC in some publications, albeit not being a fully acknowledged oncovirus. Moreover, recent microbiome studies set increasing grounds for new hypotheses on bacteriophages as interesting additional modulators in CRC carcinogenesis and progression. The present Review summarizes how particular groups of viruses, including bacteriophages, affect cells and the cellular and microbial microenvironment, thereby putatively contributing to foster CRC. This could be achieved, for example, by promoting several processes – such as DNA damage, chromosomal instability, or molecular aspects of cell proliferation, CRC progression and metastasis – not necessarily by direct infection of epithelial cells only, but also by interaction with the microenvironment of infected cells. In this context, there are striking common features of EBV, CMV, HPV and JCV that are able to promote oncogenesis, in terms of establishing latent infections and affecting p53‐/pRb‐driven, epithelial–mesenchymal transition (EMT)‐/EGFR‐associated and especially Wnt/β‐catenin‐driven pathways. We speculate that, at least in part, such viral impacts on particular pathways might be reflected in lasting (e.g. mutational or further genomic) fingerprints of viruses in cells. Also, the complex interplay between several species within the intestinal microbiome, involving a direct or indirect impact on colorectal and microenvironmental cells but also between, for example, phages and bacterial and viral pathogens, and further novel species certainly might, in part, explain ongoing difficulties to establish unequivocal monocausal links between specific viral infections and CRC.

## Graphical abstract

Latent DNA viruses disrupt the cell cycle and alter several colorectal cancer‐related regulatory proteins and signalling pathways, including the induction of mutations. Some bacteria contribute to foster a pro‐oncogenic microenvironment, further laying the ground for the establishment of CRC. Recent evidence suggests an interesting role of phages, for example, by modulating the balance between different bacterial species, and further species promoting indirect carcinogenesis.

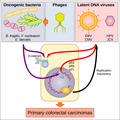

**Abbreviations**
BMMFs: bovine meat and milk factors
CD: Crohn’s disease
CDT: cytolethal distending toxin
CMV: cytomegalovirus
COX‐2: cyclooxygenase‐2
CR2: type II complement receptor
CRC: colorectal cancer
DNMT1: DNA methyltransferase 1
EBV: Epstein–Barr virus
ETBF: enterotoxigenic *B. fragilis*
FOX1: forkhead box M1
gp350: glycoprotein 350
HBoV: human bocavirus
HBV: hepatitis B virus
HCV: hepatitis C virus
HERV: human endogenous retrovirus
HFK: foreskin fibroblasts
HIV: human immunodeficiency virus
HPV: human papillomavirus
HTLV‐1: human T‐cell lymphotropic virus genotype 1
IBD: inflammatory bowel disease
IL‐6: pro‐inflammatory interleukin‐6
ITAM: immune receptor tyrosine‐based activation motif
JCV: John Cunningham virus
KGD: Lys‐Arg‐Gly
KSHV: human Kaposi sarcoma virus
LEF1: lymphoid enhancer factor 1
MHC‐I: histocompatibility complex class I
MIV: Inoue**–**Melnick virus
mtrII: morphological transforming region II
NTBF: nontoxigenic *B. fragilis*
PAMP: pathogen‐associated molecular pattern
PI3K: phosphoinositide‐3‐kinase
PML: progressive multifocal leucoencephalopathy
PP2A: protein phosphatase 2A
TAg: large T antigen
tAg: small t antigen
TCF: T‐cell factor
TERT: catalytic subunit of telomerase
UC: ulcerative colitis

## 1 Introduction

Colorectal cancer (CRC) represents the second most common type of cancer worldwide and is responsible for about half a million deaths per year [1]. The risk of developing CRC increases sharply with the age of 50, although a trend towards younger people with sporadic CRC is rising as well, and about half of the CRC patients who undergo surgery will experience recurrence, which limits life expectancy of these patients to only 5 years in average [2]. CRC develops slowly, and symptoms such as intestinal cramps or bleeding are observed only when the tumour has grown to a considerable size [3]; thus, an early detection, and the clear definition of causes and risk factors of developing CRC, is paramount.

Every person has an approximately 4% lifetime risk of developing CRC, but some factors can increase this likelihood [3, 4]. Specifically, for example, the incidence of CRC has been increasing in countries that adopted the ‘Western‐type’ diet, suggesting an association between nutritional lifestyle and the incidence of this type of cancer [5]. Chronic inflammation of the intestine is a risk factor for the development of CRC: Crohn’s disease (CD) and ulcerative colitis (UC), together known as inflammatory bowel diseases (IBDs), increase the risk of CRC to up to 40% [6, 7]. The incidence of IBD has escalated in the recent past, causing, for example, an economic burden of over US$6 billion in the United States alone [8, 9]. Another element that has been increasingly reported to boost the risk of sporadic CRC is infection [10]. Together, these risk factors indicate that microbial species, potentially modulated or even inflicted by nutritional and environmental components, inflammatory conditions or specific infections, might at least be cofactors in CRC carcinogenesis, progression or even metastasis. Recently, even a potential new class of microbial species ingested by daily Western nutrition, specifically from bovine milk, serum and dairy products, bovine meat and milk factors (BMMFs) [11], has been suggested that currently is being taxonomically placed between bacterial plasmids and single‐stranded DNA (ssDNA) viruses. Such exciting novel discoveries, which support hypotheses on the ingestion of pathogens with particular components of our nutrition, need to be analysed further to determine their exact potential causal contribution to CRC. Since data on such putatively novel species, and their taxonomic classification, are still in development, this Review will focus at summarizing the evidence on already fully characterized viral species, particularly those that have been most frequently found in, and associated with, CRC.

Seven viral taxa are currently recognized as human carcinogens: Epstein–Barr virus (EBV or human herpesvirus type 4), human papillomavirus (HPV), human Kaposi sarcoma virus (KSHV), hepatitis B virus (HBV), hepatitis C virus (HCV), human immunodeficiency virus (HIV) and human T‐cell lymphotropic virus genotype 1 (HTLV‐1) [12]. Of these, only EBV and HPV, together with cytomegalovirus (CMV or human herpesvirus type 5) and John Cunningham virus (JCV), have been consistently reported to be prevalent in CRC [13, 14, 15]. In these studies, the odds ratios (OR) between infection and development of CRC have been reported to range between 0.7 and 58.8 for HPV, 0.9 and 9.0 for JCV, 0.1 and 10.4 for CMV, and 1.0 and 4.5 for EBV [16, 17, 18, 19]. Such a wide range of relative risk estimations certainly illustrates how difficult it still is to establish a causal relationship between infection with these viruses and the development of CRC.

Moreover, recent microbiome studies have highlighted the importance of bacterial viruses (i.e. viruses infecting bacteria, better known as bacteriophages, or phages) in the aetiology of CRC [20]. Several experimental and epidemiological studies have suggested an association between the presence of certain bacteria and CRC [21, 22]. For instance, although there is a strong association between infection with *Helicobacter pylori* and the risk of gastric cancer carcinogenesis, the current consensus is that several bacterial species can contribute to CRC carcinogenesis [23, 24]. Most interestingly, due to their capability of selectively killing specific bacteria, phages most likely can play a decisive role by contributing to the alteration of the bacteriome, causing an imbalance in the intestine known as dysbiosis [25].

The present Review will not seek to outline the complete human intestinal microbiome, which would be too extensive to cover in one Review, but instead summarizes the role of particular viruses that currently have the highest level of evidence and prevalence in the carcinogenesis and progression of CRC. Towards this end, the literature presented herein was collected through a search in the PubMed/Medline database combining the medical subject heading (MeSH) terms ‘Colorectal Neoplasms,’ ‘Colorectal Cancer,’ ‘Crohn Disease,’ ‘Crohn’s Disease,’ ‘Colitis, Ulcerative,’ ‘Viruses,’ ‘Virus,’ ‘Viridae’ and ‘Bacteriophages.’ These were extended by ‘Inoviridae,’ ‘Simian virus 40,’ ‘Papillomaviridae,’ ‘Herpesviridae,’ ‘Cytomegalovirus,’ ‘Epstein‐Barr virus’ and further specific viral entity names as soon as the literature found indicated an accumulation of data on these in CRC. Our Review will discuss the epidemiological evidence linking certain viral species to this cancer entity, and the molecular pathways and mechanisms employed by these viruses that could help in fostering the insurgence, progression or even metastasis of colorectal carcinomas. In addition, we will seek to outline interesting commonalities in the molecular action between particular viral species that are reflected in the molecular landscape of CRCs, which might be indirect evidence that particular infections might have contributed to the disease.

## 2 Herpesviruses

### 2.1 EBV

Epstein–Barr virus is one of the most widespread viruses in humans, with a prevalence of about 90% in the adult population [26]. EBV is associated with over 200 000 cases of cancer per year, with a prevalence that has increased in the last two decades [27]. Moreover, EBV is responsible for about one‐tenth of all gastric cancer cases [28, 29], but a clear causative association between EBV infection and CRC is still missing [30]. Some studies failed to identify EBV in CRC sections [31, 32, 33], whereas others identified EBV in 12.8% of 274 cases of colorectal carcinoma [34] and in 5% of 19 colon adenocarcinomas [35]. EBV was observed by PCR in 19% of 186 CRC sections [36] and in 60% of 15 CRC tissues, but only in 27% of the surrounding normal tissues [37]. In our own study involving whole‐genome analyses of CRC primary tumours and in one matched corresponding metastasis [38], and in primary CRCs investigated with PCR [39], we also found EBV sequences in a comparable range of frequencies.

The infection of B lymphocytes, the main reservoir of this virus, is well characterized: EBV envelope glycoprotein 350 (gp350) binds to the type II complement receptor (CR2), which is expressed on lymphocytes, thymocytes, epitheliocytes, dendritic cells and endothelial cells. The binding between gp350 and CR2 initiates the internalization of EBV, but CR2 is also an activator of NF‐κB and determines the overexpression of pro‐inflammatory interleukin‐6 (IL‐6) [40, 41]. In addition, the attachment of EBV to the target cell activates signal transducer and activator of transcription 3 (STAT3), but the process is still not completely understood [42]. Since EBV is transmitted via saliva, it is expected that the virus should infect epithelial cells to gain access to the lymph nodes, where the primary target cells reside. However, the mechanism of infection of these cells is still poorly defined, although it is much less efficient than infection of the lymphocytes [43, 44]. The removal of the gene encoding EBV surface glycoprotein BMRF‐2, which binds integrins α3, α5, αv and β1, from the viral genome impaired the infection of epithelial cells but not B lymphocytes, suggesting that this protein must be involved in the infection of epithelial cells [45]. Interestingly, especially within the intestine and CRC, it has been reported that EBV‐derived molecules can transmit from B lymphocytes, in which EBV resides as a frequent reservoir, to epithelial cells via microvesicles [46]. These microvesicles can contain different EBV‐derived molecules, such as LMP1, one of the major EBV‐related oncogenes, or noncoding RNAs (ncRNAs; EBERs) [47, 48]. Therefore, paracrine mechanisms of triggering oncogenic changes in colorectal epithelial/CRC cells can be speculated that are brought about by intestinal B lymphocyte‐derived vesicles, especially since infected resting B lymphocytes in the gut have been reported that can be reactivated in certain instances to produce virus, or viral products, able to contribute to the malignant phenotype during an intermittent reactivation of cell growth [49, 50].

The EBV genome is a linear double‐stranded DNA (dsDNA) molecule of about 170 kb subdivided into two portions (U_S_ and U_L_), separated by inverted repeats, and bears over 100 open reading frames (ORFs) and several micro‐RNA (miRNA)‐encoding regions [26, 50, 51]. The virus initiates a period of latency, during which its expression is kept at a minimum. Immunological suppression and other environmental stimuli can induce a lytic stage that produces the viral progeny. The virus replicates differently during the lytic and the latent phases [52]. The lytic replication starts at *oriLyt*, produces linear concatemers by rolling circle amplification, and requires the viral replication machinery encoded by the viral genes *BZLF1*, *BALF5*, *BMRF1*, *BALF2*, *BBLF4*, *BSLF1*, *BBLF2* and *BBLF3* [53]. Conversely, the latent replication is initiated at *oriP* and proceeds via theta amplification. It requires the viral protein EBNA1 only and is carried out synchronously with the S phase by the host’s cellular replication machinery [54]. Since the virus is almost always latent, it strongly depends on host cell replication. Thus, EBV has evolved to modulate the cellular environment by sustaining the S phase of the cell cycle, thus maintaining in the infected cell [55].

EBV infection affects several cell signalling pathways and induces an alteration of the host genome’s methylation profiles, resulting in the abnormal expression of genes involved in cytokine regulation, cytoskeleton formation, cell proliferation and cell adhesion [56, 57, 58]. EBV infection is associated with higher levels of T‐cell factor (TCF) and lymphoid enhancer factor 1 (LEF1) transcription factors by an epigenetic mechanism that remains active even after a complete loss of the viral genomes [59]. Therefore, EBV infection has the capability to induce ‘hit‐and‐run’ mutagenesis in a cell, a process that, in theory, could contribute to CRC carcinogenesis, progression, or even metastasis. Indeed, in our own recent study [38], we found higher mutational rates to be present specifically in CRC metastatic lesions as compared to corresponding primary tumours, some with evidence of EBV‐derived sequences [39], and it remains to be investigated experimentally whether EBV infection and/or EBV‐derived molecules gained directly or indirectly from, for example, intestinal B cells are capable of contributing to such (changes of) patterns observed during CRC carcinogenesis, progression or metastasis.

The viral protein BZLF1 is a transactivator of EBV replication, and its expression marks the end of the latent phase and the induction of lytic replication [60]. BZLF1 interacts with NF‐κB and p53, enhancing the expression of IL‐10 [61, 62, 63, 64]. EBNA1 directs the replication machinery to *oriP* and tethers the viral DNA to the host’s chromosomes, ensuring the proper replication and partition of the episomes during mitosis [65]. However, EBNA1 can also be involved in the transforming process towards malignant cells. For example, it can cause chromosomal instability [66], and the transfection of mice with expression plasmids for EBNA1 resulted in a higher rate of lymphomas in the recipients [67]. EBNA1 can also enhance the expression of survivin (an apoptotic suppressor) and STAT1, while repressing TGF‐β [68, 69]. EBNA1 can bind a region located on chromosome 11 that contains several promoters, altering the expression of many genes, including *HDAC3* and *MAP3K1* [70]. Nevertheless, the role of EBNA1 is only as an accessory to transformation, since its deletion does not alter the establishment of the transformed phenotype [71].

In keratinocytes, it has been shown that EBV induces molecular alterations of the cells that, again, last even after losing the virus. In the nucleus, β‐catenin interacts with TEC/LEF1, triggering the transcription of genes involved in proliferation and motility [72]. LMP1 is known to activate STAT3 together with the epidermal growth factor receptor (EGFR) [73, 74], inhibit the activity of p53 [75], and upregulate β‐catenin by reducing its proteasomal degradation [76]. Interestingly, all of these molecules have been reported to be instrumental in CRC progression, and again, it is tempting to speculate that a virus such as EBV might be able to leave molecular imprints within also colorectal (cancer) cells, which either remain after the virus is lost from the (cancer) cell, or which remain after the cancer cell has acquired particular EBV‐derived molecules from surrounding microenvironmental cells such as B lymphocytes (see above). *In vivo* models demonstrated that LMP1 stabilizes β‐catenin by inhibiting *WTX* gene expression, whose encoded protein is part of the destruction complex [77]. However, others reported that EBV might modulate the Wnt signalling pathway in a manner not associated with LMP1 [78]. In nasopharyngeal carcinomas, LMP1 levels were positively correlated with higher expression of the transcription repressors Snail and Twist, which in turn decreased E‐cadherin levels, promoting both oncogenesis and metastasis [79, 80]. Moreover, LMP1 activated DNA methyltransferase 1 (DNMT1) expression, repressing E‐cadherin and boosting the nuclear accumulation of β‐catenin [81, 82]. As a result, the activation of NF‐κB triggered by β‐catenin upregulated the expression of survivin [83]. These Wnt/catenin‐related molecular pathways and EMT/metastasis‐related molecular pathways are essential in CRC carcinogenesis and progression. Conversely, it is still debated whether EBV can cause additional damage to infected cells through mechanisms involving, for instance, ‘hit‐and‐run’ mutagenesis or (microvesicular) exchange from microenvironmental lymphocytes.

Moreover, EBV proteins can certainly play interesting roles in immune and microenvironmental cells and thus potentially contribute to further interactions between the immune system, microenvironmental cells in general, and CRC (primary and metastasizing) carcinoma cells. The amino‐terminal domain of LMP2A contains an immune receptor tyrosine‐based activation motif (ITAM) and a proline‐rich motif (PY) that interacts with factors of the PI3K signalling pathway and ubiquitin ligases Nedd4 in B lymphocytes [84, 85]. Even in human foreskin fibroblasts (HFK), LMP2A was able to upregulate β‐catenin and boost its nuclear translocation by virtue of these motifs [86, 87]. LMP2A inhibits the destruction complex, thus stabilizing β‐catenin and promoting its nuclear localization, subsequently upregulating NF‐κB targets such as survivin in gastric carcinomas [88]. EBV subverts immune response with several strategies to sustain the infection. For instance, EBNA1 and LMP1 inhibit major histocompatibility complex class I (MHC‐I)‐mediated presentation to cytotoxic T lymphocytes [89, 90], whereas BILF1 increases the degradation of MHC‐I molecules [91]. In cytotoxic T lymphocytes, LMP2A upregulates the expression of galectin‐1, which induces apoptosis [92]. BCRF1, a viral homolog of IL‐10, reduces the production of interferon gamma (IFN‐γ) [93], whereas BZLF1 inhibits the expression of IFN‐I [94].

Such examples invite speculations as to whether similar mechanisms of interaction between colorectal (cancer) cells, immune cells and microenvironmental cells might also be active and contribute to, or modulate, CRC carcinogenesis or progression, especially since, in our own recent work, we found that, within EBV‐positive CRC samples, EBV was localized especially in lymphocytes [39]. Generally, EBV positivity of CRC samples, and EBV localization in cancer‐associated lymphocytes in addition to a few epithelial (cancer) cells, has already been reported by others as well [95, 96].

### 2.2 CMV

Cytomegalovirus is structurally very similar to EBV and has a similar replication cycle, but infects many more cell types than EBV, including epithelial cells, macrophages, dendritic cells, monocytes and fibroblasts [97]. Like EBV, CMV is also widespread in the population, but it is especially relevant during immune suppression and pregnancy, in contrast to EBV [98]. CMV is disseminated by asymptomatic hosts via several bodily fluids, such as urine, saliva and genital secretions [99]. The primary disease produced in the immunocompetent hosts is mononucleosis [100]. It is believed that the virus might reactivate without clinical signs, spreading from lymph nodes to other tissues years after the first infection [101]. In some cases, life‐threatening organ failures might arise [102]. Congenital CMV can cause severe neurological impairment in newborns [103], and circumstantial evidence links infection to atherosclerosis [104]. In the immunocompromised host, CMV can cause pneumonia, gastroenteritis, retinitis, hepatitis, leucopenias (which contributes to opportunistic infections), organ failure and death; it is also a major concern for organ transplantation [101].

Epidemiological evidence connecting CMV infection to CRC is not as strong as for EBV, but there is both epidemiological and molecular corroboration of CMV oncogenic activity in other types of tumours, such as medulloblastoma, breast and ovarian cancer [105]. A recent systematic review reported a 2‐ to 5‐time higher CMV infection rate in gastric cancer patients than controls [106].

A previous meta‐analysis reported that CMV infection is associated with an OR of 6.6 of developing CRC [107]. Conversely, a survey of 65 colorectal adenomas and 65 colorectal adenocarcinomas by immunohistochemistry failed to identify CMV in these tissues [108, 109]. However, it has been reported that formalin processing might hamper the detection of CMV within tissues; thus, PCR is a more sensitive approach for prevalence analysis [110]. Accordingly, 11% of 56 formalin‐fixed paraffin‐embedded CRCs were found to be positive for CMV DNA by PCR [111]. Nonetheless, CMV has been recovered in CRC sections. For instance, an early study identified CMV in 57% of seven colon tumours [112], another in 78% of nine colorectal polyps and in 92% of 12 adenocarcinomas, but not in the surrounding non‐neoplastic colon biopsy [113]. Quantitative PCR confirmed that CMV DNA can be more frequently recovered in CRC tissues than in the normal adjacent tissue [110, 114]. Metagenomic analysis of colon tissues reported the presence of EBV and CMV in at least one IBC patient [115]. Also, in our own studies of colorectal primary tumours and matched metastases [38], we found evidence for CMV sequences after Blast filtering in one‐sixth of the primary colorectal carcinomas [39]. It has been reported that CMV infects stem‐like mutants of the CRC‐derived HT29 cell line with higher efficiency [116], a feature that suggests that the interaction between CMV and intestine‐derived host cells is still not completely understood and might influence viral detection.

Like EBV, CMV also hampers the functions of several cellular factors. CMV infection of fibroblasts is followed by the accumulation of cyclin E, p53, pRb and β‐catenin [117, 118]. Despite high p53 expression levels, p53 activity is impaired by CMV because the protein is sequestered within nuclear replication foci [119]. Moreover, the morphological transforming region II (mtrII) viral protein represses the transactivating activity of p53 [120]. CMV promotes angiogenesis and cell proliferation in the context of an improved wound healing [121, 122]. EMT, an important molecular and cell morphological switch found in the transition from cancerous to metastatic cells, is also more frequent during CMV infection [123]. CMV impairs the proper assembly of the DNA repair machinery, avoiding initiation of the blockage of replication that follows DNA damage, thus extending the S phase [124]. Interestingly, the effect of CMV on the Wnt signalling pathway is still not completely understood, but some aspects appear to be opposite to that of EBV [125]. Infection of human dermal fibroblasts and human placental trophoblasts with CMV resulted in decreased levels of β‐catenin due to higher rates of protein degradation in the presence of unchanged transcription rates [126]. Also, CMV stabilized tankyrases (also known as PARPs) in HFK cells, resulting in higher axin expression that, in turn, degraded β‐catenin [127]. Still, all of these molecules play essential roles in CRC carcinogenesis and progression, and the observation of CMV detection in CRC tissues as described above should promote functional studies as to which exact molecular roles a CMV infection can play in colorectal (cancer) cells.

As to further microenvironmental interactions, CMV promotes a pro‐inflammatory environment that can foster DNA damage and oncogenic progression. The host responds to CMV infection by producing IFN‐γ, IL‐6 and IL‐10 (all of which promote inflammation), and by an accumulation of cytotoxic T cells [128]. Markers of inflammation, such as cyclooxygenase‐2 (COX‐2), are overexpressed in cells infected by CMV [129, 130]. At the same time, it has been shown that CMV infection stimulates the production of superoxide O_2_^–^ (which can cause DNA damage) by macrophages, IL‐6 and IL‐10 (both of the latter boosting the expression of STAT3) [131, 132, 133]. Again, it needs to be investigated how exactly CMV might promote CRC carcinogenesis by stimulating these and further microenvironmental interactions; nevertheless, clinical and epidemiological observations on particular inflammatory conditions that pose a significantly higher risk for carcinoma development, and especially of COX‐2 inhibitors being protective against colon carcinogenesis [134], suggest the need to perform more specific analyses to link CMV infection with CRC.

## 3 Papillomaviruses

The epidemiological evidence connecting HPV to CRC is controversial [135]. Certainly, there is a known causative association of HPV with anal carcinoma, which ontologically is a totally different entity: this virus has been retrieved in 96.7% of 366 anal cancers [136] and 54.5% of 11 anal biopsies [137]. In contrast, some studies failed to observe HPV DNA in CRC tissues [138, 139, 140, 141, 142, 143], but others detected HPV in 42.2% of 45 CRC biopsies [144] and in 51% of 55 CRC tissues [145]. The prevalence of HPV in CRC tissues has been reported to have a mean value of 41.7%, as compared to the 32.0% in the adjacent normal tissues in a review article in 2011 [146].

Although mutations of p53 are a CRC landmark, CRC tissues infected with HPV often show an intact *Tp53* gene. Yet, the functionality of p53 in CRC cells is disrupted, leading to the suggestion that HPV inactivates p53, thus promoting cancer [16]. In addition, more and more experimental evidence links HPV infection to a pro‐oncogenic modulation of the Wnt/β‐catenin pathway [147]. For example, in oropharyngeal cancer, tumour cells infected with HPV expressed higher β‐catenin than HPV‐negative tumours [148]. Furthermore, the nuclear localization of β‐catenin was higher in HPV‐positive tumours than HPV‐negative control tumours [149], a characteristic also confirmed in tonsillar tumours [150]. The nuclear import of β‐catenin was mediated by the viral proteins E6 and E7, since miRNA knockdown by synthetic miRNA targeting the E6/E7 promoter resulted in a decrease in nuclear, and an increase in membrane‐associated, β‐catenin [149].

The genomes of papillomaviruses consist of covalently closed circular dsDNA molecules subdivided into two main transcriptional units – early (E) and late (L) – each of them containing a promoter (P_E_ and P_L_, respectively) and a polyadenylation site [151]. The viral protein E2 modulates viral transcription in concert with other cellular transcriptional factors [152]. This modulation is fundamental in maintaining the expression of the appropriate amount of viral proteins to sustain the S phase without prompting the cell into G_0_, which would imply the termination of DNA synthesis [153]. Unlike herpesviruses, papillomaviruses do not encode a DNA polymerase; thus, the virus completely depends on the host cell’s S phase for its replication. Maintenance of this phase is obtained by the action of the viral proteins E7, E6 and E5, which are all oncoproteins.

HPV infection can induce a switch in the biochemistry of the infected cell, which is interesting in the context of the fact that diets rich in sugar, and metabolic conditions causing obesity in general have been observed to be associated with an increased risk for CRC [154, 155]. Towards this end, E7 can modify the structure of M2 pyruvate kinase, boosting glycolysis and reducing the cell’s dependence on oxygen [156]. It is known that cancer cells increasingly rely on glycolysis rather than oxidative phosphorylation (Warburg effect) [157, 158]. Thus, HPV provides a selective advantage for infected cells to survive in hypoxic conditions [159], an ability that we hypothesize would be able to support cancer cell survival in the growth of especially metastatic lesions as well, since they are frequently known to harbour a hypoxic centre, especially when they reach a particular size [160, 161]. Remarkably, hypoxia upregulates p53 [162]; therefore, the capability of HPV to inactivate this protein provides a further advantage to cancer precursor cells.

E7 targets members of the retinoblastoma (Rb) family of proteins (pRb, p107 and p130), leading to their degradation and liberating transcription factor E2F from these inhibitors [163, 164]. E7 is transported into the nucleus independently of its interaction with pRb [165, 166]. Mutations in the domain binding to pRb do not abrogate this protein’s transforming activity, indicating that E7 can contribute to carcinogenesis at multiple levels within a cell [167]. For instance, E7 can bind to cyclins A, D and E in the absence of pRb [168, 169], and inhibits the dephosphorylation of protein kinase B, which is carried out by protein phosphatase 2A (PP2A), thus maintaining the activity of the phosphoinositide‐3‐kinase (PI3K) signalling pathway, which is one of the important signalling axes in CRC [170, 171]. Again, PP2A is also an inhibitor of the Wnt pathway as an essential hallmark of CRC [172], and HPV infection can affect the Wnt/β‐catenin signalling pathway several‐fold. Towards this end, ectopic expression of E6 and E7 is associated with a decreased expression of Siah‐1 proteins, E3‐ubiquitin ligases that interact with the destruction complex that degrades β‐catenin [173]. Interestingly, a few years ago our group showed that a network of miRNAs significantly deregulated in CRC metastases (one of them being miR‐210, which is inducible by hypoxia; see above) downregulated Siah‐1 as well; thus, this particular action of E6 and E7 would add to a similar molecular axis of CRC progression and metastasis [174]. In anal carcinoma (which is considered to be a significantly different carcinoma entity than CRC, however), the expression of the miRNAs miR‐16, miR‐20a, miR‐150 and miR‐155 did not significantly vary between anal squamous intraepithelial lesions and normal tissues [175]. Cells expressing high levels of the genes *CCNA2*, *CCNB1*, *CCNB2*, *MSH6* and *MCM7* (all involved in the regulation of cellular proliferation) also expressed high levels of miR‐15b [176]. Such a ncRNA can be under the transcriptional regulation of E2F also [177], suggesting that the overexpression of E2F due to the activity of the viral oncoprotein E7 [178] is able to increase the transcription of miR‐15b. Since miR‐15b stimulates cell proliferation through the expression of CCNA2, CCNB1, CCNB2, MSH6 and MCM7, an induction of this ncRNA by HPV can have severe consequences on the cellular environment [176]. In recent years, it has become additionally evident that HPV can transcribe ncRNAs [179]. Still, however, although the involvement of HPV‐encoded ncRNA in fostering several types of squamous cell carcinomas (cervical, head and neck, and oropharyngeal) has been established, viral ncRNA in the genesis of CRC is still poorly understood [180].

Nevertheless, in the context of CRC‐related important pathway molecules, HPV protein E6 has been shown to bind to β‐catenin with the PDZ domain, thus promoting nuclear localization of β‐catenin [181]. Expression of E6 stabilizes β‐catenin [182] and also enhances the expression of forkhead box M1 (FOX1), increasing the rate of nuclear translocation for β‐catenin [183]. E7 also binds to histone deacetylases and the E2F suppressor p21, overall extending the S phase [184, 185]. The consequences of this action can be several‐fold: for example, E7 induces chromosomal instability (which is often detected in CRC) and an increased rate of integration of foreign DNA into transfected cells [186, 187]. Furthermore, the unscheduled extension of DNA replication activates p53, triggering apoptosis [188]. HPV has evolved to neutralize p53 by encoding E6, which forces p53 into ubiquitin‐dependent degradation [189, 190, 191]. E6 also induces overexpression of the catalytic subunit of telomerase (TERT), further promoting DNA replication [192]. E6 interferes with many other cellular proteins involved in ubiquitination (E6AP), apoptosis (Bak, c‐myc, procaspase 8, FADD, survivin, TNF), transcriptional regulation (CBP/p300, E6TP1, hADA3, Gps2, tuberin), immune surveillance (IRF‐3, TLR9), chromosomal stability (hMCM7, XRCC1, O(6)‐methylguanine DNA methyltransferase), differentiation (ERC‐55, fibulin, paxillin, zyxin), and cell adhesion and proliferation (hDlg, MAGI‐3, hScrib, MUPP1, MAGI‐1, MAGI‐2, PATJ, PTPN3) [193]. Finally, HPV protein E5 enhances the functions of both E6 and E7 and inhibits the degradation of activated EGFR, sustaining signalling pathways promoted by this receptor [194, 195]. This and further molecular targets of HPV described in this paragraph are evidently essential players in CRC carcinogenesis, progression and/or metastasis, and could be, in theory, ideal mediators of HPV‐promoted carcinogenesis and/or progression in this context.

## 4 Polyomaviruses

John Cunningham virus (also known as JCPyV) was first isolated from brain tissues of a patient with progressive multifocal leucoencephalopathy (PML) [196]. Early studies on the prevalence of JCV failed to identify this virus within CRC, but such a failure might have been due to the structure of the viral genome. Subsequent work, involving DNA digestion with topoisomerase and genome amplification with degenerated primers, showed a prevalence of JCV of 81–89% in CRC tissues, and the virus was enriched in CRC sections with respect to the surrounding normal tissues [197, 198, 199]. Even when the virus was present in both tumour and healthy tissues, the viral load was reported to be significantly higher in the former (10^3^–10^4^ copies per µg of tissue) than in the latter (10^1^–10^2^ copies·µg^−1^) [200].

John Cunningham virus establishes a life‐long and asymptomatic infection of the kidneys in about half of the adult population [201]. JCV also infects tonsillar stromal cells, bone marrow cells, oligodendrocytes and astrocytes [202]. In the presence of immunosuppression, the virus can reactivate, causing PML [203]. PML is usually fatal, and its incidence has drastically increased with the spread of acquired immunodeficiency syndrome (AIDS) [204]. Given the role of immune suppression in PML and the high prevalence of JCV infections, the use of immunosuppressive therapy to treat CD has raised concern about potential side effects [205].

The JCV genome is a circular dsDNA molecule of 5 kb in length [206]. Replication of the virus depends on the large T antigen (TAg), which recruits the cellular replication machinery and functions as a helicase [207]. Alternative splicing of the TAg ORF produces additional regulatory proteins (small t antigen, T´_135_, T´_136_, T´_147_, T´_152_ and T´_165_) [208]. There are two primary forms of JCV: the prototypical and the rearranged. The former is the full‐genome length commonly recovered from urine and renal tissues, possibly representing the viral infectious entity [209]. The latter shows rearrangements in the genome, particularly in the regulatory region, and several versions have been recovered in different cell lines. This suggested that the rearrangements represent adaptations to specific cell types [210]. Remarkably, a subvariant of the JCV strain, Mad‐1 (M1∆98), has been consistently recovered in CRCs but not in normal surrounding colorectal tissues [211]. The introduction of either Mad‐1 or M1∆98 into the colon carcinoma cell line RKO revealed direct interactions between TAg and both p53 and β‐catenin, and the infected cells developed chromosomal instability and loss of contact inhibition [212]. Other studies have confirmed that TAg interacts with β‐catenin, promoting its stability and nuclear import [213]. Moreover, TAg binds to Rac1, which in turn connects with β‐catenin, resulting in further stabilization of the latter [214]. The β‐catenin stabilization carried out by TAg, together with the aforementioned epidemiological and experimental data, renders JCV as a chief suspect in fostering carcinogenesis and potentially also progression of CRC [17].

Further epidemiological and experimental evidence on TAg itself confirmed the hypothesis of a central role of this protein in CRC. TAg was observed in 77% of CRCs as compared to 72% of healthy surrounding tissues, and its expression was associated with increased chromosomal instability and hypermethylation of tumour suppressor genes [215]. Others have reported the presence of TAg in 26% of CRC sections but in none of the normal colon tissues [216]. TAg interacts with pRb and p53, extending the S phase and replication of the viral genome [217]. Furthermore, TAg stimulates ataxia telangiectasia mutated (ATM) and ataxia telangiectasia mutated and Rad3‐related (ATR), triggering G_2_ arrest and boosting viral amplification [218].

Further JCV proteins disrupt the cell cycle. Small t antigen (tAg) and the short isoforms T´ inhibit pRb [219, 220]. Moreover, tAg inhibits PP2A, which dephosphorylates agnoprotein (AP), a JCV regulatory protein [221]. As in the case of HPV E7, this interaction might indirectly promote the stability of β‐catenin, activating the Wnt pathway. Moreover, AP represses the expression of p21 and impairs the double‐strand break (DSB) repair mechanism of infected cells [17, 222]. This is interesting in the light of our own recent finding that AC3 mutational signatures, which reflect mutations/malfunctions in DSB repair and BRCAness, were increasingly ongoing and active in progressive CRCs, and specifically in metastatic lesions [38]. Again, it is tempting to speculate that such findings of mutational genomic signatures might be, at least in part, an imprint of particular viruses, in this case, JCV, that could have aided in CRC carcinogenesis and/or metastasis.

**Fig. 1:**
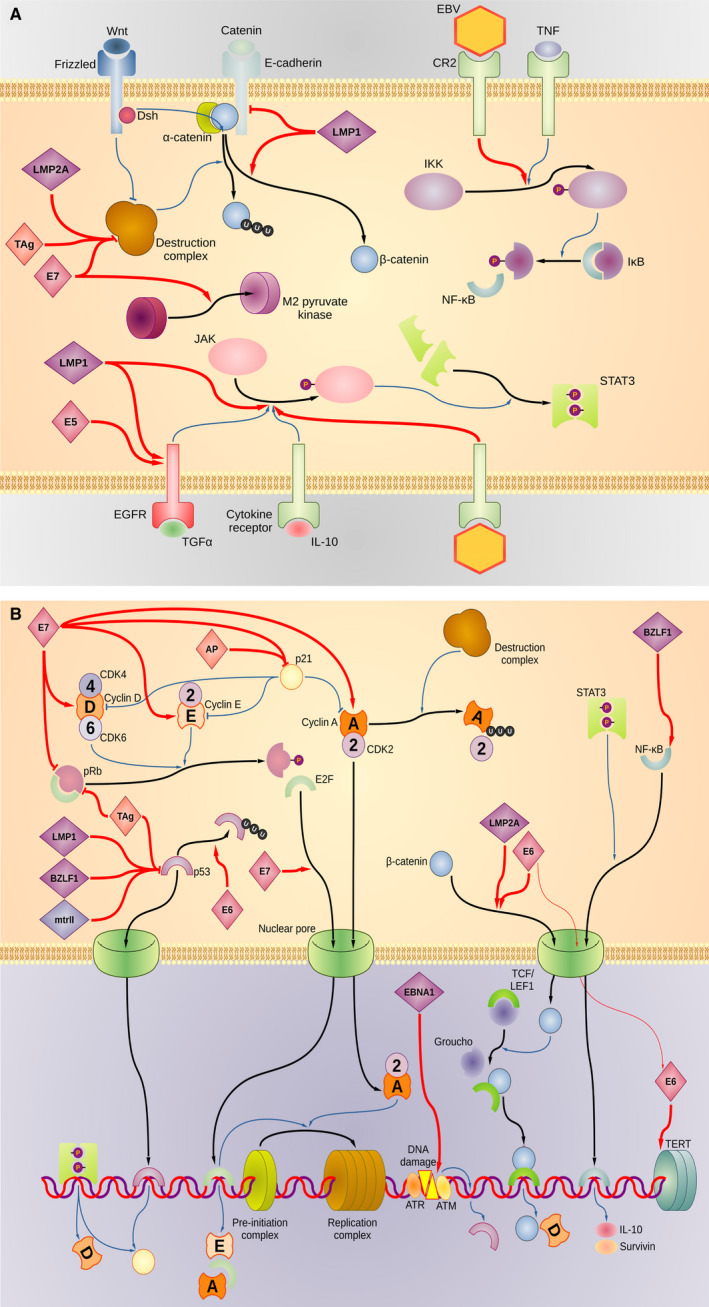
(A) Major signalling molecules and pathways by which viruses discussed in this review could contribute to CRC carcinogenesis and progression: Involvement of cell surface receptors and cytosolic signalling molecules. Oncogenic viruses sustain their multiplication by hijacking the cellular replication machinery and have coevolved to target the same cellular pathways to extend the S phase and avoid senescence or apoptosis. The cellular multifunctional protein β‐catenin is targeted at several levels. In nonstimulated cells, β‐catenin is sequestered at the membrane in a complex with E‐cadherin, and any unbound β‐catenin is rapidly targeted for ubiquitination by the destruction complex, which is formed by several proteins, including APC and axin. Binding of the mitogen Wnt to the frizzled receptor conjugated with Dsh results in inhibition of the destruction complex and release of β‐catenin from cadherin. JCV, HPV and EBV repress activity of the destruction complex via TAg, E7 and LMP2A, respectively. In addition, LMP1 decreases the degradation of E‐cadherin while promoting the stability of β‐catenin. The binding of the mitogen TNF to the receptor CR2 induces the phosphorylation of IKK, which in turn phosphorylates IκB and the release of the transcription factor NF‐κB. The binding of EBV to CR2 activates this process independently from TNF. The transcription factor STAT3 is involved in sustaining cell proliferation and is formed upon phosphorylation carried out by JAK. Activation of JAK is also dependent upon phosphorylation induced by the cytokine receptor and EGFR. The former is activated upon binding with TGF‐α and the latter by the association with IL‐10. Binding of EBV to the cytokine receptor activates JAK. Furthermore, EBV promotes STAT3 activity by encoding LMP1, which inhibits the degradation of EGFR and stimulates JAK. HPV E5 also activates EGFR. In addition, HPV E7 forces a structural change in the M2 pyruvate kinase, promoting glycolysis and reducing the cell’s biochemical dependence on oxygen. It is important to note that virus‐derived molecules such as EBV‐derived LMP1 might also enter (cancer) cells via, for example, microvesicles from microenvironmental (in the EBV case B) cells in a ‘paracrine’ fashion, not only by direct infection of the cell. (B) Major signalling molecules and pathways by which viruses discussed in this review could contribute to CRC carcinogenesis and progression: Involvement of nuclear import and intranuclear events. The transcription factor E2F is central in the expression of genes involved in converting the prereplication complex into the fully formed replication machinery. This transition is tightly regulated by cyclins and pRb. In particular, pRb sequesters E2F, and the release of E2F is promoted by cyclins D/E and inhibited by p21. JCV and HPV affect this process by promoting the degradation of pRb via TAg and E7, respectively. Furthermore, E7 facilitates the nuclear transportation of E2F and enhances the activity of cyclins A/D/E. Cyclin A is instrumental in the formation of the replication complex, but this cyclin is ubiquitinated by the destruction complex. As shown in Fig. 1A, the destruction complex is inhibited by TAg, E7 and LMP2A. The suppressor p21 inhibits cyclins A/D/E, but it is counteracted by JCV‐derived AP and HPV‐derived E7. S phase extension activates effectors of DNA damage response such as p53. Viral replication associated with direct DNA damage activates the kinases ATM and ATR, which stimulate the expression of p53. EBV‐derived EBNA1 has been reported to cause direct DNA damage. JCV, HPV, EBV and CMV prevent blockage of the S phase by targeting p53 via TAg/tAg, E6 (which induces the ubiquitination of p53), BZLF1/LMP1 and mtrII, respectively. Intranuclear NF‐κB activity is enhanced by EBV‐derived BZLF1, whereas the transcription factor STAT3 facilitates its nuclear translocation. The protein β‐catenin is involved in the activation of cellular proliferation by releasing the inhibitor Groucho from the transcription factor TCF/LEF1. Nuclear import of β‐catenin is enhanced by HPV‐derived E6 and EBV‐derived LMP2A. HPV facilitates DNA replication by upregulating the expression of TERT through the action of E6.

In summary, there is epidemiological, but also, at least in part, some molecular evidence that supports hypotheses for classically known DNA viruses, such as CMV, HPV, EBV and JCV, to be potential contributing factors to CRC. A graphical summary of the main mechanisms that could be targeted in this context is given in Fig. 1A,B.

## 5 Phages

Dysbiosis has been recognized as a risk factor in CRC development, since about one‐third of CRC patients show dysbiosis and, at least in part, associated inflammation [223, 224]. Faecal transplantation of mice with bacterial communities found in CRC patients resulted in CRC development in the recipients, highlighting the risk associated with specific bacteria within the microbiome, such as *Bacteroides* [225]. Using metagenomic approaches, recent work has shown differences in the prevalence of phages between the healthy and inflamed intestine [226]. Specifically, an increased richness (number of species) of phages belonging to the order of *Caudovirales* has been consistently reported [227]. Although it is understood that phages can modulate human physiology at several levels, the scale of this influence is still not well comprehended [228]. It has been proposed that phages might alter the overall balance of the bacteriome by targeting species, which are not necessarily pathogenic *per se*, promoting the expansion of driver bacteria (those capable of causing inflammation) and passenger bacteria (those contributing to oncogenesis), and thus CRC carcinogenesis and/or progression [229, 230]. In addition, phage‐induced bacteriolysis releases cellular debris into the microenvironment, which can induce inflammation. For instance, bacterial DNA and lipopolysaccharides act as a pathogen‐associated molecular pattern (PAMP) that trigger an immune response [231].

The current status of knowledge suggests that the role of phages in CRC is indirect. Although it is known that phages can get access to epithelial cells [232], the current hypothesis rather is that they do infect human cells *sensu proprio* and do not encode for oncoproteins. However, by promoting the growth of pathobionts, able to damage intestinal cells and to establish local inflammation, phages represent a risk factor for CRC, whose importance has only recently started to be appreciated [233]. It has been proposed that phages are capable of inducing a ‘leaky gut’, loci of increased intestinal permeability that facilitate the infiltration of pathogenic bacteria and foster chronic inflammation [234]. Thus, phage‐mediated carcinogenesis implies the presence of pathogenic and/or oncogenic bacteria. Several bacterial species have been suggested to be involved in this process, but the most recurrent in the literature are *Escherichia coli*, *Bacteroides fragilis*, *Enterococcus faecalis* and *Fusobacterium nucleatum*.

*Escherichia coli* is a commensal of the human gut, and strains such as *Nissle 1917* are commonly used as probiotics [235, 236]. However, some strains contain genes encoding virulence factors that can damage intestinal cells, causing chronic inflammation and, subsequently, cancer [237]. One of these virulence factors is cytolethal distending toxin (CDT) (which is also present in strains of *Shigella, Campylobacter, Actinobacillus* and *Helicobacter*), which causes DNA damage and blocks exposed cells within the G_2_/M transition [238]. Colibactin is another virulence factor that damages the DNA of exposed cells and is common among *Enterobacteriaceae*, including *E. coli* [21, 239]. Colibactin is encoded by a 54‐kb genomic region, known as polyketide synthases (*psk*) island, which promotes the synthesis of nonribosomal peptides [240]. Remarkably, even the probiotic strain *Nissle 1917* contains the *psk* island [241], highlighting how our understanding of the bacterial role in inflammation and cancer still needs to be refined with more intense functional studies, especially regarding strains widely applied as probiotics in an, up to now, rather uncritical fashion. Furthermore, in our own analyses of primary colon tumours and liver metastases [38], we observed sequences of several coliphages specifically in these tissues but not in normal matched colon tissues [39], and it remains to be functionally determined how this additional phage level of complexity contributes to the overall picture of microbiome‐associated modulation of CRC carcinogenesis or metastasis.

*Bacteroides fragilis* can be subdivided into two main groups: nontoxigenic and enterotoxigenic. The former (NTBF) is used as a probiotic, but the latter (ETBF) can cause tumours in mice, and it is associated with CRC in humans. ETBF asymptomatically infects about one‐third of the human population and produces a toxin (a metalloprotease known as BFT) that induces chronic inflammation by transactivating STAT3 [242]. BFT binds to E‐cadherin and mediates its cleavage, with the consequent release of β‐catenin into the cellular milieu [243].

*Enterococcus faecalis* is also used as a probiotic and shows anti‐inflammatory activity. However, even in this case, certain strains have been suggested to be involved in the carcinogenesis of CRC [244, 245]. The peculiarity of this bacterium is that it does not encode for a cytotoxic virulence factor, but produces extracellular superoxide (·O_2_^–^), which stimulates the COX‐2 pathway in macrophages that is responsible for damaging epithelial cells [246, 247]. Although the metabolic process that generates ·O_2_^–^ is still poorly understood, pathogenic strains produce a higher amount of it than commensal strains, suggesting that this molecule provides a selective advantage for *E. faecalis* [248].

*Fusobacterium nucleatum*, which is commonly encountered in the oral cavity, activates macrophages. It has been shown that *F. nucleatum* infection increases the infiltration of macrophages into gingivae [249]. Furthermore, this bacterium stimulates macrophage migration and conversion into the activated M1 phenotype *in vitro* and *in vivo* [250, 251]. This opportunistic bacterium is an endocellular parasite able to gain access to epitheliocytes using the E‐cadherin CDH5, which is recognized by the bacterial surface protein FadA, resulting in higher cytoplasmic expression of β‐catenin [252]. Autoinducer 2 (AI‐2) is a secretory protein involved in the quorum‐sensing response, and it is shared by different bacterial families [253]. AI‐2 produced by *F. nucleatum* upregulates the production of IL‐1β, promoting the transition of macrophages into the M1 phenotype [254]. *Fusobacterium nucleatum* increases the expression of NF‐κB [255] and encodes a two‐subunit immunosuppressive protein (FIP) of about 10 kDa that arrests T lymphocytes in G_0_, thus triggering immunosuppression [256]. To the extracellular space, *F. nucleatum* releases short peptides (formyl methionyl alanine and leucyl‐phenylalanine) and fatty acids (butyrate, propionate and acetate) that are chemo‐attractants for immunosuppressive cells [257].

If lytic phages selectively target commensal species within the intestine, the results could expand opportunist bacteria, such as the aforementioned, that lead to chronic inflammation [258]. Indeed, in our own recent genome sequencing studies of CRC primary tumours and corresponding metastases [38], we found evidence for the presence of sequences of different Enterobacteria, Bacillus, Streptococcus and other phages [39]. The prolonged exposure of epithelial cells within the colon and rectum to an inflammatory environment ultimately results in the accumulation of genetic damages within these cells. In this context, phages play a more decisive role than being simple modulators of bacterial populations. Phages give their hosts a selective growth advantage, and it has been observed that antibiotic treatment boosts a horizontal genetic exchange between bacteria and phages [259]. It is known that phages can cross the intestinal barrier by transcytosis of epithelial cells [232], although potential effects that phage particles might exert on the cellular environment are still not, or poorly, understood [231]. For example, an exposure of several cell lines, including lymphocytes and fibroblasts, to several types of phages (T2, MS2 and ϕX174) did not cause an increase in mutation rates or chromosomal aberration of the exposed cells, although phage T2 inhibited the DNA replication of the treated cells [259]. Interestingly, especially in the context of CRC and the microbial flora of the human intestine, phage T4 was shown to reduce the rate of phagocytosis of *E. coli* and of reactive oxygen species (ROS) production in phagocytes in *in vitro* models [260]. Also, *in vivo*, T4 curtailed antibody production in mouse models [261]. The attachment of phages to the membrane of lymphocytes and epithelial cells has been demonstrated to suppress cellular amplification [260]. These results even suggest that, against the current hypothesis of phages as merely indirect players in CRC oncogenesis or progression, phages such as T4 or coliphages might be exerting an additional, more active, role in colorectal inflammation and cancer. This is supported by the observation that phages can cause a misfolding of cellular proteins, and the debris generated by the lysis of bacterial cells can trigger an immune response causing conditions in the host, which raises the case of considering phages as human pathogens *tout court* [231]. Further functional and molecular studies need to support this hypothesis.

**Fig. 2:**
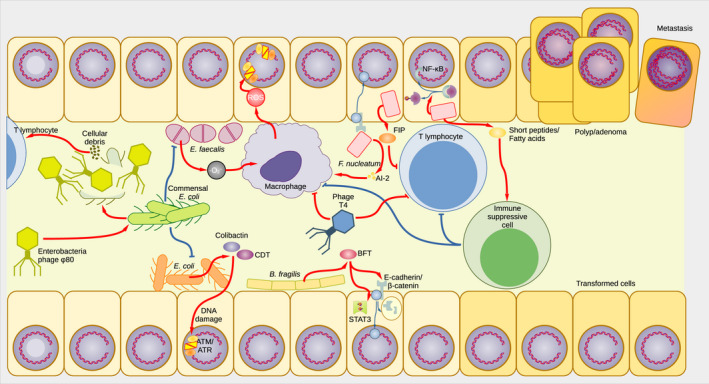
Bacterial and phagial influence on the colorectal environment. Some bacteria can induce cell damage and immunological imbalance able to contribute to fostering CRC. Certain strains of *E. coli* bear virulence factors encoding colibactin and CDT, capable of inducing DNA damage in colonocytes. *E. faecalis* produces superoxide (·O_2_^–^) as a by‐product of its metabolism. Superoxide stimulates macrophages to produce RSO that also induces DNA damage in colonocytes. Virulent strains of *B. fragilis* encode BFT that promotes cell proliferation by activating STAT3. Furthermore, BFT promotes the degradation of E‐cadherin that, in turn, increases β‐catenin levels. *F. nucleatum* is an endogenous parasite that stimulates cell proliferation by activating the E‐cadherin, with the subsequent release of β‐catenin in the cytoplasm and its nuclear translocation. *F. nucleatum* also promotes the activation of NF‐κB and secretes molecules (AI‐2) that induce activation of macrophages. Moreover, *F. nucleatum* modulates the immune system by releasing FIP that inhibits T lymphocytes involved in clearing the infection. *F. nucleatum* also releases short peptides and fatty acids that activate immune suppressive cells, further weakening the immune response to infection and extending the bacterium’s activity. Pathogenic bacteria usually are contrasted by the commensal bacteria present in the intestine. In the figure, such an interaction is represented by a commensal *E. coli* species downregulating both *E. faecalis* and a pathogenic *E. coli* strain. However, in real conditions, there would be a network of species cross‐regulating each other. Bacteriophages can be important players to alter this equilibrium by, for example, targeting the commensal species and promoting the proliferation of the pathogenic species. The targeting and lysis of bacteria cause cellular debris, which, in itself, can trigger immune responses [231]. The latter two scenarios are exemplified in the figure by Enterobacteria phage ϕ80. Besides, phages are able to modulate the immune system, avoiding a deleterious immune response. For instance, it has been proposed that T4 binds to T lymphocytes [260], thus inducing immune suppression. In certain conditions not fully understood, this type of immune suppression can promote the proliferation of pathogenic bacteria. The combination of bacterial interaction and phage modulation provokes genetic damage and chromosomal instability within colonocytes, which can result in a transformed phenotype, potentially leading to polyps, adenomas and, eventually, (metastatic) carcinoma cells.

Still, phages are also able to provide beneficial features to the human body. Phages modulate the activity of macrophages, dendritic cells, lymphocytes, monocytes and granulocytes, reducing the expression of IL‐2, TNF‐α and IFN [261]. It has been proposed that phages containing the sequence Lys‐Arg‐Gly (KGD) in their capsids attach to activated T lymphocytes, causing immunosuppression [262]. Such modulation is beneficial because it reduces the risk of abnormal immune responses. In addition, phages can help fight infections by lysing invading bacteria. It has been suggested that phages constitute a sort of primordial adaptive immune response residing in the mucus surrounding the intestine, removing foreign bacteria before they can reach the epithelial cells underneath [263]. We think that, especially according to, for example, environmental circumstances (e.g. the intestinal microenvironment and individual microbiome, nutritional factors, pH, potentially also environmental toxins which are actively, or unknowingly, ingested), phages might take on opposing roles, as either ‘friends’ or ‘foes’, which facilitate the spread of opportunistic bacteria. Taken together, it is highly likely that it is the context that determines the outcome of the phagial influence on human physiology, inflammation and cancer. A graphical summary of some of the functions of particular phages in the intestine, within dysbiosis and as potential contributors to CRC carcinogenesis and progression, is given in Fig. 2.

## 6 Other viruses

The virus families discussed so far have the highest epidemiological evidence for a potential association with CRC. A few more have been implicated, and it will be highly interesting in the future to perform more studies on these, epidemiologically and functionally. With an exciting review a few years ago, Harald zur Hausen suggested a link between the consumption of red meat of certain species and the development of CRC [264]. Specifically, a limited group of viruses [papillomaviruses, polyomaviruses and torque teno virus (TTV)] are able to resist the temperature of cooking and survive in the meat, thus potentially explaining the increase in CRC incidence in countries whose population consumes, or started to consume, high quantities of red bovine meat [265]. Papillomaviruses and polyomaviruses, as described in the preceding paragraphs, do have a connection with CRC. TTV (family *Circoviridae*) was originally isolated from a hepatitis patient (whose initials were T.T.) in the late 1990s [266, 267] and was subsequently observed at high frequency in CRC tissues by de Villier *et al*. (38 out of 50 sections) [268]. TTV was present in the blood of 150 out of 153 (98.0%) CRC patients compared with 43 out of 50 control subjects (86.0%) [269], and in 72 out of 82 (87.8%) CRC tissues compared with 16 out of 40 (40.0%) normal colon sections [270]. TTV has been reported to infect, asymptomatically, a large proportion of the human population [271, 272] and has been associated with hepatitis, pulmonary diseases, inflammation and systemic lupus erythematosus [273]. Interestingly, in our genome analysis [38], we can confirm the presence of TTV in raw genomic data of CRC, including some liver metastases [39], although of course these data still need to be considered with caution since it is known that TTV is highly prevalent in the blood in general [264], and that the liver is one of the organs with the highest content, and flow, of blood within the human body.

Other viruses that have rarely been reported to have been detected in CRC are human bocavirus (HBoV) and Inoue–Melnick virus (IMV) [19]. HBoV (family *Parvoviridae*) was isolated from children with respiratory infections in the mid‐2000s [274] and then observed in the blood of healthy donors [267]. HBoV was reported in 9 out of 44 (20.5%) [275] and 24 out of 101 (23.8%) [276] CRC sections, but also as low as one out of 66 CRC patients (1.5%) compared with one out of 91 healthy controls (1.1%) [277]. IMV (no assigned family) was isolated from a multiple sclerosis patient in the 1980s [278] and later isolated from five CRC‐derived cell lines (SW‐480, WiDr, LoVo, DLD‐1 and SW‐1463) [279]. Serological investigation in 26 CRC patients showed the presence of IMV in all of them, compared with 9 out of 26 (34.6%) cases of non‐CRC and 10 out of 26 healthy controls (38.5%) [280], and, in another sample set, in 20 out of 24 CRC cases (83.3%) [281]. IMV was present in 100% of nine CRC samples but absent in ten normal colon tissues from healthy controls [279]. Up to now, no functional link has been published, as far as we know, to CRC.

Human endogenous retroviruses (HERVs) belong to a family of proviruses of retroviral origin that constitute about 8% of the human genome [282]. Virtually all HERVs are inactive, but a small proportion can express their viral genome if certain, still not completely understood, conditions are met [283]. The expression of HERV oncoproteins has been associated with oncogenesis [284, 285, 286]. High expression of HERV ERV3‐1 has been reported in CRC samples [287], and transcriptome analysis in one paper suggested that an increase in HERV transcripts was associated with a poor prognosis in CRC patients [288]. Also, in our own series of colorectal cancer/metastasis samples [38], we frequently observed HERV‐K113 sequences in the genomes; however, due to the setting of the analysis (whole‐genome sequencing), we could not determine whether there were differences in retroviral expression between normal tissues, primary colorectal tumours and metastases, and therefore, future studies on this might lead to additionally interesting discoveries [39].

As mentioned in Introduction, HIV is also a recognized oncogenic virus, which has primarily been associated with lymphomas. However, due to its immunosuppressive capabilities, it is also involved in facilitating the establishment of chronic infections of other viruses known to progress to cancer, such as HPV [289, 290, 291]. Conversely, the relationship between HIV infection and CRC risk is less clear, although it is increasingly recognized in general that HIV‐positive patients have a higher cancer risk and a poorer prognosis than HIV‐negative people [292, 293]. HIV‐positive people have a hazard risk of 2.3 for cancer as compared to HIV‐negative people, specifically 2.8 for pancreatic cancer. The hazard risk is 3.0 for HCV infection and CRC, and 2.4 for an HIV/HCV coinfection and CRC [294]. The exact oncogenic mechanisms that link HIV to CRC are not clearly understood yet. One hypothesis proposed that this association is not due to the depletion of T4 lymphocytes, but rather due to the side effects of the antiviral drugs [295]. Interestingly, anti‐HIV drugs were effective in the treatment of CRC and associated metastases [296]. For instance, raltegravir (RAL) inhibits HIV integrase but also Fascin‐1, a marker of metastasis [297]. Similarly, zidovudine is a nucleotide analogue that interferes with HIV reverse transcriptase and is able to restore the sensitivity to cisplatin in resistant cancers. Thus, it has been used in combination with 5‐fluorouracil to treat metastatic CRC [298]. Tenofovir, another agent used against HIV, has given promising results in the treatment of adenocarcinoma in rats recently [299].

Finally, chimeric plasmid‐like structures formed by sequences derived from bacteria (*Acinetobacter*) and ssDNA viruses (*Geminiviridae* and *Nanoviridae*), named bovine meat and milk factors (BMMFs), were reported recently by de Villier, zur Hausen *et al*. [11] in bovine meat, serum and cow milk, as already indicated in Introduction. BMMFs were subsequently confirmed in CRC tissues, and the current hypothesis is that they act as indirect carcinogens via an interaction with (intestinal) macrophages, inducing chronic inflammation and the damage of DNA [300]. Another aetiological agent in the form of a self‐replicating plasmid associated with red meat was associated with transmissible spongiform encephalopathy, named Sphinx [301]; thus, BMMF and Sphinx might be the same entity. Further analysis reported a high similarity between Sphinx and *Acinetobacter* [302], and the transfection of Sphinx into embryonic kidney cells (HEK293) indicated an alteration in cellular expression that could support a pathogenic role for this entity [303]. It remains to be seen in future studies how these novel taxa might contribute functionally to CRC carcinogenesis, progression or metastasis.

## 7 Conclusions and perspectives

Despite the differences in structure and replication strategies, the (onco‐)viruses associated with direct carcinogenesis most frequently observed within CRC (EBV, CMV, HPV, JCV), and discussed herein, share some similarities:

- They are all DNA viruses that establish latent infections.
- They depend on the replicating machinery of infected cells.
- They disrupt the cell cycle, especially by targeting p53, pRb and also p21.
- They interfere with EMT‐/EGFR‐associated pathways.
- Especially – and this is supported by the most abundant pieces of evidence – they target the Wnt/β‐catenin pathway in multiple ways.

Moreover, a putative involvement of phages in the CRC context is an increasingly exciting field, which nevertheless renders the microbial contribution to CRC carcinogenesis more complicated. Still, some of the mechanisms of carcinogenesis carried out by oncogenic bacteria as discussed above, which in turn can be modulated by specific bacteriophages, show similarities with the viral ones and, in particular, again highlight the importance of the Wnt signalling pathway as a specific target of pro‐oncogenic colorectal commensals in CRC progression.

The disruption of the Wnt/β‐catenin pathway is particularly intriguing since a significant number of mutations, and further genomic changes that have been reported in CRC, are concentrated within this signalling system (besides others, such as p53‐associated pathways, which are also viral targets). This we have seen confirmed in our own work of whole‐genome sequencing of CRC primary tumours and corresponding metastatic lesions [38]. It is striking that, in CRC, the frequency of, for example, APC mutations is 50–85% but only 7% in bladder carcinoma, and 0.7% in pancreatic adenocarcinoma [304, 305, 306]. Assuming that the rate of genetic mutations is the same for all cells, it is clear that specific mutations are being selected in certain tumour entities. Certainly, it is acknowledged that the presence of mutations is a potentially necessary, but not sufficient, cause of carcinogenesis. For instance, the mouse strain Min (multiple intestinal neoplasia) bears a mutation in the *APC* gene that disrupts the protein, and therefore, it is a model for familial adenomatous polyposis in humans [307]. Although the mutation is present in all of the mouse cells, tumours appear in the intestine only, indicating that the intestinal cells are more sensitive to Wnt signalling pathway disruptions. Still, the processes that promote mutational selections in natural CRC carcinogenesis are still not entirely clear. Given the molecular impact particular viruses can exert on, for example, not only the Wnt but also other pathways, such as DNA DSB repair (AC3 mutational pattern according to Alexandrov *et al*. [308, 309]), a mutational pattern we, and subsequently others, found to be increasingly pronounced in CRC progression towards metastasis [38, 310], we consider it tempting to suggest that particular viruses as discussed in this Review could have imposed particular mutational/genomic/molecular patterns into CRC cells. This might have occurred even if their presence in particular tumours or colorectal tissues was nonpermanent, or if particular virus‐derived molecules might have found their way into CRC cells not by direct infection, but in ‘paracrine’ manners from microenvironmental cells (e.g. EBV). Thus, increasing mutational patterns within, for example, DNA DSB repair might, at least in part, be due to the actions of, for example, passenger viral infections or of reboosted infections residing in, for example, intestinal lymphocytes that transfer viral pro‐oncogenic molecules. The fact that molecular imprints could have remained, even if the virus infection is not evident or active any more, could be one explanation as to why it might be particularly difficult to establish clear and unequivocal epidemiological correlations between specific viral infections such as EBV, CMV, HPV or JCV, and CRC carcinogenesis and progression. Factors of indirect carcinogenesis by (in part nutritionally modified or inflicted) phage‐bacterial interactions and further novel species (e.g., BMMFs) most likely at least add to such a scenario. This certainly is a mere hypothesis at the moment and would need more experimental work. Still, our additional recent genomic observation that apolipoprotein B mRNA‐editing enzyme, catalytic polypeptide (APOBEC) signatures were enriched in some CRC primary tumour and corresponding metastasis samples [38] is supportive of this hypothesis, since APOBEC enzymes, amongst other activities, have specific functions in the defence against viral infections [38, 311, 312]. Also, our recent finding, confirmed by others, that the *MACROD2* gene was most frequently hit by structural variations (SVs) in CRC metastases is interesting in this context, since it becomes increasingly clear that the family of MARylating PARPs exerts functions in host–virus interactions, limiting viral replication [313, 314].

Finally, it is still a matter of debate why the oncogenic viruses discussed in the present Review have been established as causative for disease much more clearly in other types of cancer than in CRC. For instance, no biological evidence is able to explain why EBV is a recognized risk factor in gastric cancer but not in CRC. The specific cellular environment, and its difference between gastric and intestinal environmental conditions, is probably involved in generating a different response to viral infection. For instance, it is known that the gastric pH is extremely acidic (between 1 and 4), which raises to a pH between 7 and 8 in the small intestine, with highly different microbial populations in the stomach, large and small intestine [315]. It is possible that EBV could access the host cell via two independent mechanisms: one pH‐independent through direct fusion with the plasma membrane, and another mediated by endocytosis that is more effective at low pH [316, 317]. The acidity of the environment could be therefore one parameter affecting the infectivity of particular viruses and, consequently, their oncogenic potential or their general ability to interact as players within a particular microenvironment. Alternatively, it is possible that the distinct microbial communities present in the stomach, large and small intestine might pose specific competitive arrangements that alter virus infectivity. Furthermore, gastric and intestinal epithelial cells most certainly have slightly different genetic expression landscapes that could profoundly affect viral activity.

Clearly, more experimental work combining viral and host (organ) analysis is needed to achieve a more complete understanding of how viruses contribute to carcinogenesis and cancer progression in different carcinoma entities. If they arise in organs that show complicated and multiple parameters of environmental exposure, for instance via the lumen of the digestive tract, this endeavour certainly will pose high levels of challenges to the community of basic molecular and medical scientists within fields as different as (molecular) oncology, microbiology, nutritional and veterinary sciences, environmental sciences, and others, and require complex interdisciplinary efforts. Nevertheless, this challenge for the near future will undoubtedly be a highly exciting one.

## 8 Take‐home messages

- CRC represents the second most common type of cancer worldwide, and its incidence is predicted to increase in the next years, also affecting increasingly younger people.
- Currently, the evidence is highest for EBV, CMV, HPV and JCV for an association with CRC.
- EBV, CMV, HPV and JCV are all DNA viruses that establish latent infections and rely on the cellular replication machinery.
- EBV, CMV, HPV and JCV disrupt the cell cycle, altering the Wnt/β‐catenin pathway in multiple ways, and act at further (CRC‐associated) pathways.
- Further, novel infectious agents need to be investigated in future functional/epidemiological studies as to their function in CRC.
- Bacteriophages are gaining increasing awareness for a putative role in CRC carcinogenesis or even progression, due to their ability to modulate the intestinal microflora and the immune system.

## Conflict of interest

The authors declare no conflict of interest.

## Author contributions

Both LM and HA contributed ideas and wrote the review. HA prompted the idea of reviewing this topic, including novel ideas on phages and further species such as BMMFs, and their potential roles in CRC.
